# Sustainable Extraction Methods Affect Metabolomics and Oxidative Stability of Myrtle Seed Oils Obtained from Myrtle Liqueur By-Products: An Electron Paramagnetic Resonance and Mass Spectrometry Approach

**DOI:** 10.3390/antiox12010154

**Published:** 2023-01-09

**Authors:** Angela Fadda, Paola Montoro, Gilda D’Urso, Nicoletta Ravasio, Federica Zaccheria, Daniele Sanna

**Affiliations:** 1 Institute of the Sciences of Food Productions, National Research Council, Traversa La Crucca, 3, 07100 Sassari, Italy; 2 Department of Pharmacy, University of Salerno, Via Giovanni Paolo II 132, 84084 Fisciano, Italy; 3 Institute of Chemical Sciences and Technologies “G. Natta”, National Research Council, Via Golgi 19, 20133 Milano, Italy; 4 Institute of Biomolecular Chemistry, National Research Council, Traversa La Crucca, 3, 07100 Sassari, Italy

**Keywords:** green chemistry, metabolomics, LC-MS, electron paramagnetic resonance spectroscopy, food waste, multivariate data analysis

## Abstract

Myrtle liqueur production generates high amounts of by-products that can be employed for the extraction of bioactive compounds. Bio-based, non-toxic and biodegradable solvents (ethyl acetate and 2-methyltetrahydrofuran), and a mechanical extraction were applied to myrtle seeds, by-products of the liqueur production, to extract oils rich in phenolic compounds. The oils obtained were characterized for yield, peroxide value (PV), lipid composition, and total phenolic concentration (TPC). The phenolic profile of the oils, determined by LC-MS, the antioxidant activity, and the oxidative stability were also analyzed. A validated UHPLC-ESI-QTRAP-MS/MS analytical method in multiple reaction monitoring (MRM) mode was applied to quantify myricetin and its main derivatives in myrtle oils. The results pointed out clear differences among extraction methods on myricetin concentration. The oxidative stability of myrtle oils was studied with electron paramagnetic resonance (EPR) spectroscopy highlighting the effect of the extraction method on the oxidation status of the oils and the role of phenolic compounds in the evolution of radical species over time. A principal component analysis applied to LC-MS data highlighted strong differences among phenolic profiles of the oils and highlighted the role of myricetin in the oxidative stability of myrtle oils. Myrtle oil, obtained from the by-products of myrtle liqueur processing industry, extracted with sustainable and green methods might have potential application in food or cosmetic industries.

## 1. Introduction

Food, pharmaceutical, and cosmetic industries are continuously searching for biologically active natural compounds to meet the consumer demand for all-natural products. The concept of “natural product” is often linked to that of environmental sustainability. Consumer awareness towards environmental problems, such as hazardous chemicals disposal and waste management, including food waste, has significantly increased during the last few decades. Health and the environment now direct the choices of consumers, so it is becoming essential to develop sustainable and green strategies to obtain natural biomolecules from agri-food side streams and by-products. The main goal of green chemistry is to replace hazardous chemicals with safer and renewable ones, reducing, at the same time, energy consumption [1]. The choice of the proper solvent at the industrial level is extremely important since it affects the process efficiency, the amount and the type of biomolecules extracted, and the safety of the final extracts. In vegetable oils and fats, the benchmark solvent for extraction is *n*-hexane for its ease of use and removal by evaporation [2]. The drawback of *n*-hexane is its toxicity and origin from fossil sources. It has been calculated that the extraction of vegetable oils releases in the atmosphere more than 20 million kg of hexane, making this solvent a hazardous air pollutant [3]. To replace *n*-hexane, other solvents with biodegradable properties, no- or low-toxic activity, and greener features have been suggested for the extraction of lipids for food and non-food applications. Among them, 2-methyltetrahydrofuran and ethyl acetate have been employed for lipid extraction in several oil rich seeds [2,4,5]. Moreover, these solvents, having a medium polarity, have the advantage of extracting both polar (polyphenols) and non-polar compounds (lipids) at the same time [6,7]. The walnut oil extracted with ethyl acetate yielded a high linoleic acid content and had a good amount of polyphenols [7]. Similarly, on black cumin seeds, the extraction with 2-methyltetrahydrofuran provided an oil with high linoleic acid, tocopherols, and total phenolic content [8].

The agri-food chain generates annually 198.9 kg/year of waste pro capita, mainly in developed countries [9]. The management of food waste is one of the biggest concerns for processing industries. In the last few decades, a growing body of literature is contributing to change the role of food processing by-products from waste to resource [10,11,12]. By-products and waste are now perceived as promising and useful sources of potentially valuable bioactive compounds that could find application in the food chain or in the cosmetic industry according to the principles of circular economy and sustainable development [9].

The recovery of oil and phenolic compounds from grape seeds obtained from the by-products of the vinery industry is a relevant example of the exploitation and valorization of food waste that could be extended to other food industry bio-residues [10,11].

Myrtle is an evergreen shrub widely appreciated for the antiseptic, anti-inflammatory [13], antimicrobial, and antioxidant properties [14,15,16] of its leaves and berries. The seeds are a good source of nutritionally essential polyunsaturated fatty acids (PUFA) that have a concentration of linoleic acid of about 77% [17]. In Europe, the myrtle plant is mostly associated with the liqueur produced from its berries through a hydro-alcoholic infusion process. The myrtle industry annually generates a huge amount of by-products made up of the berries (pulp and seeds) left after the extraction process. At present, myrtle by-products are considered waste or are underexploited as a combustible material, even if they still have a lot of biologically active molecules like phenolic compounds and fatty acids [18,19] that might be employed by food or cosmetic industries. The attempts to exploit myrtle by-products carried out so far have concerned a zoo-technical use to feed animals [20] and a nutraceutical use based on their antioxidant and antiaging effects [13]. As far as we know, a green extraction method to obtain seed oil has never been applied to myrtle by-products.

The EPR spectroscopy coupled with the spin trapping method has been applied to study the oxidative stability of several oils subjected to accelerated storage conditions or to thermal treatments [21,22,23,24]. Most of the studies has been performed on stripped oils to study the evolution of lipid radicals or on oils enriched with antioxidants to evaluate their protective effect during oil storage or thermal treatments [25,26]. The presence of antioxidants hinders the propagation step of the lipid peroxidation slowing down the formation of the radical adducts formed by the reaction of lipid radicals with *N-tert*-butyl-α-phenylnitrone (PBN), which, being relatively stable, can be detected with EPR. The effect of endogenous antioxidants on the evolution of radical formation and how they affect the kinetic of lipid radical formation has been little studied.

In this paper, green extraction methodologies, based on biodegradable solvents, and a mechanical extraction have been applied for the recovery of oil from the seeds obtained from by-products of the myrtle liqueur production. The oils extracted with the green solvents ethyl acetate and 2-methyltetrahydrofuran and with the mechanical extraction process were compared with that obtained with *n*-hexane. The effect of the extraction method on oil quality was studied by a chemometric approach based on analysis of total phenols and phenolic profile, determined by LC-MS analysis, peroxide values, esterified and free fatty acids profile, and oxidative stability assessed with EPR spectroscopy.

## 2. Materials and Methods

### 2.1. Chemicals

All reagents and solvents were of analytical grade unless otherwise specified and used without further purification. The chemicals *n*-hexane (HX), ethyl acetate (EtOAc), 2-methyl tetrahydrofuran (2-MeTHF), PBN (*N-tert*-butyl-α-phenylnitrone), myricetin, myricetin 3-O-β-D-galactopyranoside, myricitrin (myricetin-3-O-rhamnopyranoside) at purity level of 99%, and ammonium thiocyanate were purchased from Sigma-Aldrich (Milan, Italy). Formic acid and methanol for extraction were purchased from VWR international PBI S.r.l. (Milan, Italy). Acetonitrile, water, and formic acid (all of LC-MS grade) were purchased from Merck (Milan, Italy). Ultrapure water was prepared using a Milli-Q system (Millipore Corporation, Billerica, MA, USA).

### 2.2. Plant Material

Myrtle seeds were obtained from the by-products of myrtle liqueur production, kindly provided by a local liqueur processing industry. Upon arrival at the laboratory, they were inspected to remove spoiled biomasses then air dried. The pulp and the seeds were separated with a laboratory seed air cleaning machine and seeds were stored until oil extraction.

### 2.3. Myrtle Oil Extraction

Myrtle oil was extracted from seeds by mechanical and chemical extraction procedures. For mechanical extraction, 100 g of seeds were put in a screw press. Oil samples were centrifuged at 3018× *g* for 15 min to remove the solid residues. Oil was separated, weighed to calculate oil yield, and stored at −20 °C until analysis.

The chemical extraction of myrtle oil was accomplished with a soxhlet apparatus by extracting myrtle seeds with *n*-hexane for 6 h at reflux until the feedstock was completely defatted. The organic solvent was evaporated under vacuum. The soxhlet extraction was compared to cold extractions carried out with HX, EtOAc, or 2-MeTHF. EtOAc and 2-MeTHF were chosen as solvents for their ability to extract both phenolic compounds and oils due to their medium polarity and for their environmental sustainability. For the extraction, 30 g of milled seeds were mixed with 300 mL of HX, EtOAc, or 2-MeTHF and left under continuous stirring for 24 h at room temperature [27]. The extracts obtained were filtered to remove solid residues, and the organic solvent was evaporated under vacuum. The oils recovered were weighed and oil yield was calculated as percentage (*w*/*w*) of oil over seed weight. Three separate extractions were performed for each extraction method. Oils obtained were stored at −20 °C and analyzed for fatty acid composition, concentration of phenolic compounds, antioxidant activity, and oxidative stability with the EPR spin trapping method.

### 2.4. Myrtle Oils Characterization

#### 2.4.1. Fatty Acids Composition

The chemical composition of myrtle oil fat was determined with gas chromatography using an HP-6890 instrument (Agilent Technology, Milan, Italy) equipped with a flame ionized detector using a non-bonded, bis-cyanopropylpolysiloxane (100 m) capillary column at isotherm T = 190 °C. Before injection, the samples (10 mg in 1 mL of *n*-hexane) were transesterified with a 2 M KOH/methanol solution (0.200 mL). The sample solution and the potassium hydroxide solution were manually shaken in a vial for five minutes, and after settling, the upper phase was injected for analysis. The assignment of the signals was done on the basis of previous works [28] and by the comparison with literature data about myrtle oil [17]. The mol % of each component was derived from the corresponding peak area.

#### 2.4.2. Determination of Peroxide Value

Peroxides were determined according to Shantha and Decker [29]. Briefly, about 10 mg of myrtle oils were mixed with 9.8 mL of chloroform-methanol 7:3 (*v*/*v*) and vortexed, then 50 µL of ammonium thiocyanate solution (394 mM) and 50 µL of a FeSO_4_·7H_2_O (18 mM) were mixed. After 15 min, the absorbance was measured at 507 nm with a Perkin-Elmer Lambda 35 spectrophotometer. Peroxide value was expressed as micro-equivalents of oxygen·g^−1^ of oil based on a calibration curve built using FeCl_3_·6H_2_O as Fe(III) source (Fe(III): 6.1 × 10^−5^–4.6 × 10^−4^ M; R^2^ = 0.99).

### 2.5. Analysis of Phenolic Compounds and Antioxidant Activity

#### 2.5.1. Extraction of Phenolic Compounds

The phenolic compounds were extracted from myrtle oils obtained with mechanical, cold HX, EtOAc, or 2-MeTHF extractions. Phenolic extracts were prepared according to Hrncirik and Fritsche [30]; 2.5 g of oil were solubilized with 5 mL of *n*-hexane. The phenolic compounds were extracted by mixing the oil solution in *n*-hexane with 5 mL of a methanol solution (MeOH/water: 60/40). The mixture was shaken for 30 min then centrifuged at 2200× *g* for 10 min. The polar fraction (bottom phase) was collected and used to assess the total phenolic compounds (TPC) concentration and to identify the phenolic compounds with LC-ESI-Orbitrap-MS and LC-ESI-Orbitrap-MS/MS analysis.

#### 2.5.2. Total Phenolic Concentration

The concentration of total phenolic compounds was determined according to the Folin Ciocalteu method, and the results were expressed as mg caffeic acid ·g^−1^ of oil (Caffeic acid 0.05–0.4 mg·mL^−1^; R^2^ = 0.99) according to Rabadán, et al. [31]. To start, 200 µL of the phenolic extract were mixed with the Folin reagent (0.5 mL); then, after 3 min, 1 mL of sodium carbonate at 35% was added. The mixture was brought to volume (10 mL) and left in the dark for two hours. The absorbance was read at 750 nm with a Perkin Elmer lambda 35 spectrophotometer.

#### 2.5.3. LC-MS Identification of Phenolic Compounds

##### Sample Preparation

The phenolic extracts, obtained as described in paragraph 5.1, were diluted in 900 µL of water (of LC-MS grade) to obtain a final concentration of approximately 1 mg·mL^−1^ and then filtered through Millex-HV Syringe Filter Unit, 0.45 µm, PVDF, 33 mm. For LC-MS analysis, 10 µL were used. Each replicate sample of the phenolic extract was analyzed in technical triplicate.

##### LC-ESI-Orbitrap-MS and LC-ESI-Orbitrap-MS/MS Analysis

LC-ESI-Orbitrap-MS analyses were performed as described by D’Urso, et al. [32]. A HPLC method coupled with a hybrid mass spectrometer, combining a linear trap quadruple (LTQ) and an Orbitrap mass analyser, was developed for the study of the main metabolites’ characteristic of the berry seeds. Experiments were performed on a Thermo Scientific liquid chromatographer based on a quaternary Accela 600 pump and an Accela auto sampler, hyphenated with a linear orbitrap hybrid mass spectrometer (LTQ-Orbitrap XL, Thermo Fisher Scientific, Bremen, Germany) equipped with an electrospray ionization (ESI) source. Chromatographic separation of metabolites was performed on an XSelect CSH C18 (Waters) column (2.1 mm × 150 mm particle size 3.5 µm). Column temperature was maintained stable at 40 °C. After optimization, the mobile phase used for the final experiments was a gradient made of solvent A (water acidified with 0.1% formic acid) and solvent B (acetonitrile acidified with 0.1% formic acid). A linear gradient program at a flow rate of 0.200 mL·min^−1^ was used: 0–15 min, from 10 to 20% (B); 15 to 25 min, from 20 to 40% (B); and 25 to 35 min, from 40 to 60% (B) then to 100% (B) for 5 min and back to 10% (B) for the other 5 min. The mass spectrometer was operated in negative ion mode. ESI source parameters were as follows: capillary voltage −12 V; tube lens voltage −121.47 V; capillary temperature 280 °C; and Sheath and Auxiliary Gas flow (N_2_) 30 and 5 (Arbitrary Units), source voltage 5 kV. MS spectra were acquired with full range acquisition covering *m/z* 200–1600. A data dependent scan was performed to obtain MS/MS experiments; corresponding precursor ions were selected as the most intensive peaks in LC-MS analysis. Spectral characteristics fragmentations allowed for the identification of phenolic compounds in addition to specific retention time by comparing with literature data and specific databases available online (MS bank, KNApSAcK). Xcalibur software version 2.1 was used for instrument control, data acquisition, and analysis.

##### Determination of Myricetin and Its Derivatives by UPLC–ESI-QTrap-MS/MS Analyses in MRM (Multiple Reaction Monitoring) Modality

To optimize the parameters for the analysis, full scan ESI-QTrap-MS and Collision Induced Dissociation (CID), ESI-QTrap-MS/MS analyses of standards were performed on an ABSciex (Foster City, CA, USA) 6500 QTrap spectrometer. The analytical parameters were optimized by infusing a standard solution of each metabolite (1 mL·min^−1^ in methanol) into the source at a flow rate of 10 L/min. Data was acquired in the negative ion MS and MS/MS mode.

Quantification of myricetin and its derivatives was performed with a Shimadzu Nexera LC system in line with the QTrapMS equipped with a kinetex EVO C18 column (Phenomenex) (100 × 2.1 mm, i.d. 1.7 µm). Mobile phases consisted of water containing 0.1% formic acid (solvent A) and acetonitrile containing 0.1% formic acid (solvent B). Column temperature was maintained stable at 40 °C. Chromatographic separation was achieved with the following gradient elution profile at a flow rate of 0.3 mL·min^−1^: 0–10 min a linear gradient 5%–100% B; and then back to 5%B for 3 min. The 6500 Qtrap spectrometer was configured for an IonSpray operation, and the compounds were detected using Multiple Reaction Monitoring (MRM) in negative ion mode. Values of additional QTrap parameters are as follows: curtain gas (CUR) = 35 (arbitrary units); collision gas (CAD) = medium; IonSpray voltage (IS) = −4500 V; temperature (TEM) = 350 °C; ion source gas 1 (GS1) = 25 (arbitrary units); ion source gas 2 (GS2) = 25 (arbitrary units). Declustering Potential (DP), Entrance Potential (EP), Collision Energy (CE), and Collision Cell Exit Potential (CXP) values were determined by infusing selected standards into the 6500 QTrap and adjusting the values to obtain the maximal response. The dwell time for each analyte was 20 ms. The total cycle time was kept to ∼0.3 s, which typically gave at least 14 points across all chromatographic peaks. Data acquisition and processing were performed using Analyst software 1.6.2 (ABSciex, Foster City, CA, USA).

Quantitative method was validated according to EMA guidelines [33]. Each standard solution was analysed in triplicate to obtain calibration curves with correlation values in the range 0.997–0.999.

The limit of detection (LOD) and the limit of quantification (LOQ) were determined with a serially diluting standard compounds analysis performed until the results of signal-to-noise ratio (S/N) reached the values of 3:1 and 10:1. The recovery and precision were adequate; the recovery rates range was from 95% to 105% within the same day.

#### 2.5.4. Radical Scavenging Activity of Myrtle Oils

The radical scavenging activity of myrtle oils was determined with the DPPH method according to Fadda, et al. [34] with slight modifications. About 10 mg of oil were solubilized in ethanol, then a fixed volume (1.9 mL) was mixed with a DPPH solution 1 mM and stored in the dark at room temperature for 30 min. The absorbance was read at 517 nm with a Perkin Elmer lambda 35 spectrophotometer. Results were expressed as µmol trolox·g^−1^ of oil. All measurements were performed in triplicate.

### 2.6. Thermal Treatment and EPR Spin Trapping Analysis of Myrtle Seed Oils: EPR Settings and Spectra Acquisition

A Bruker EMX spectrometer operating at the X-band (9.4 GHz) equipped with an HP 53150A frequency counter and a variable temperature unit ER 4111 VT was used to measure the radical adducts during oils thermal treatment. Spectra were acquired with Bruker WinEPR Acquisition Version 4.33 and simulated with Bruker WINEPR SimFonia Version 1.26. The EPR instrument was set under the following conditions: modulation frequency 100 kHz, modulation amplitude 0.106 mT, receiver gain 5 × 10^4^, microwave power 20 mW (which is with the ER 4119HS cavity, below the saturation limit), resolution 1024 points, sweep time 167.772 s, and a time constant and conversion time 163.84 ms. The selected values of time constant, sweep time, resolution, and sweep width allowed us to resolve the narrowest line corresponding to 0.049 mT.

Five µL of a 2.5 M PBN solution in absolute ethanol were dried under a nitrogen flow to avoid any interference of ethanol during the spin trapping experiment. Then, 100 µL of oil were mixed with the solid PBN, transferred into a flat cell (inner diameter of 2.5 mm, exposed oil surface 5 mm^2^, with a 25 mm terminal flat part at the bottom), and inserted into the resonant cavity heated at 90 °C. EPR spectra were get every 5 min for 4 h under continuous heating at 90 °C. The intensity of the PBN-adduct was estimated from the double integration of the spectra and was plotted against time.

### 2.7. Statistical Analysis

A multivariate data analysis was performed to evaluate the effect of extraction method on myrtle oils. LC-MS raw data of the 24 samples (3 samples for each extraction method replicate in technical duplicate) deriving from LC-ESI-Orbitrap-MS analysis (negative ion mode) were analyzed using a platform independent open source software package called MZmine (http://mzmine.sourceforge.net/; accessed on 25 May 2022). Using this toolbox with the normalization of total raw signal, 1650 peaks were detected. After exporting the processed data in tabular format (.cvs file), further analysis of the data matrix was performed with SIMCA P+ software 12.0 (Umetrix AB, Umea, Sweden) by Principal Component Analysis (PCA). PCA was performed by applying the peak area obtained from LC-MS analysis [32,35]. Pareto scaling was applied before multivariate data analysis. After evaluating that the model was representative because it gave high R2X(1) and R2X(2) value (>80% of the variance), the set of LC-MS data was integrated with Total phenolic, Total Peroxide, DPPH, and EPR data, giving a final model in which the first component describes 78.49% of variance while the second only 10,71%. The choice of principal components was realized on the basis of the fitting (R2X) and predictive (Q2X) values for the PCA model. Results of peroxides, total phenols, fatty acid composition, and radical scavenging activity were compared with a one-way ANOVA, calculating means separation with Tukey’s test or *t*-test at *p* ≤ 0.05. Statistical analysis was performed with GraphPad Prism8 for Windows software (GraphPad Software Inc. La Jolla. CA92037, USA). Three replications were performed for each analysis.

## 3. Results and Discussion

### 3.1. Oil Yield and Peroxide Values of Myrtle Oils

Myrtle oil yield is affected by the extraction method applied (Table 1). The soxhlet extraction method with *n*-hexane yielded about 9 g of oil/100 g of seeds, in agreement with Wannes and Marzouk [17] who reported a yield of about 8.9% in the extraction of myrtle seeds with the soxhlet method using petroleum ether as a solvent. This is the first time that different solvents and methods have been compared for the extraction of myrtle oil from seeds. Previous papers on other oil seeds highlighted the effect of the extraction method on oil yield [8,36]. The mechanical extraction method (ME) yielded a lower amount of oil than solvent extraction regardless of the solvent employed. The percentage of oil extracted with *n*-hexane at room temperature (about 20 °C) was similar to the soxhlet hexane extraction, indicating that the effect of temperature on extraction efficiency was counterbalanced by the increase in the extraction time. The 2-MeTHF produced a greater amount of oil compared to the other solvents; however, no statistical difference could be found with cold *n*-hexane extraction. The high extraction efficiency of the 2-MeTHF has been highlighted in previous works where even a species-dependent effect was observed. In basil seed, the extraction with the 2-MeTHF and *n*-hexane gave similar oil yields, whereas in cumin seeds, the extraction with 2-MeTHF was more effective than that with *n*-hexane [8]. The EtOAc recovered an amount of oil considerably lower than *n*-hexane and 2-MeTHF (Table 1). Such a low oil extraction efficiency, observed in other oil-bearing seeds as well, is maybe due to its polarity that limits the solubility of the lipids.

Peroxide value (PV) is an indicator of the quality and the oxidative status of an oil measuring the primary oxidation products. The PV depends on several factors associated with the unsaturation degree of the oil, the oxygen availability, and temperature. The extraction method significantly affected the PV. Mechanical and cold *n*-hexane extractions provided oils with similar PV, whereas oils extracted with EtOAc and 2-MeTHF had significantly higher PV. This difference is likely due to their high polarity that increases the amount of extracted peroxides. If compared to the most common edible oils like sunflower or extra virgin olive oils [37], myrtle oils, regardless of the extraction method, have PV beyond the limits set by WHO and FAO for edible oils (10 milliequivalents oxygen·kg^−1^ oil). However, it should be considered that, in general, crude oils before the refining process have a high PV that decreases at each refining step [38]. Moreover, myrtle biomasses employed in this work were not fresh but underwent an ethanol/water extraction process for the production of the myrtle liqueur that lasts 30–40 days and can certainly increase the PV in comparison with the oil obtained from fresh berries.

### 3.2. Fatty Acids Composition of the Oils

Table 1 reports the fatty acid composition of mechanical and solvent extracted myrtle seed oils. Regardless of the extraction method, myrtle oils had a similar fatty acid composition. Polyunsaturated Fatty Acids (PUFA) represented by linoleic acid (C 18:2) account for about 77% of total fatty acids (TFA). The four extraction methods yielded oils with very similar amounts of PUFA, although some statistical differences were observed. ME oils had the highest linoleic acid concentration (about 78%).

Oleic acid, a monounsaturated fatty acid, represents about 8% of the total fatty acids. In a previous work on myrtle fatty acid composition, oleic acid represented about 11% of TFA [17]. Palmitic acid was the main saturated fatty acid as previously reported by Wannes and Marzouk [17], followed by stearic acid that represents about 3% of the total fatty acids. The ratio of SFA to PUFA was about 0.14 in all the oils regardless of the extraction method employed. Despite sharing a similar concentration of MUFA and PUFA, myrtle oils significantly differ for the concentration of free fatty acids (FFA) (Table 1).

### 3.3. Oils Phenolic Compounds and Antioxidant Activity

#### 3.3.1. Oil Total Phenols and Antioxidant Activity

**Figure 1:**
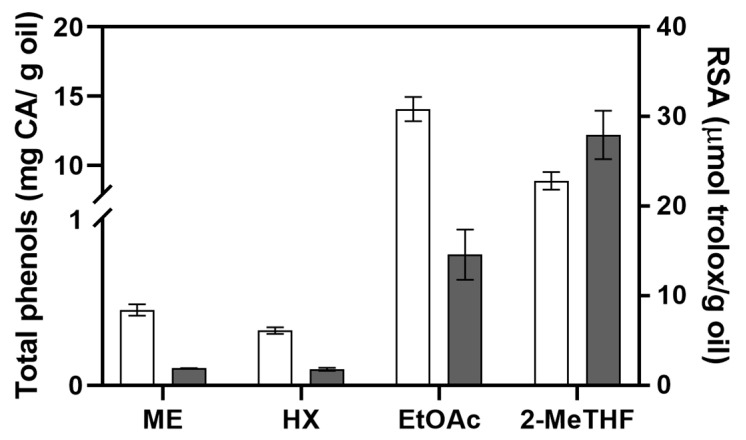
Concentration of total phenolic compounds (white bars), expressed as mg caffeic acid·g^−1^ of oil, and Radical Scavenging Activity (RSA) (grey bars), expressed as µmol trolox·g^−1^ of oil, of myrtle seed oil extracted with cold pressed extraction (ME), *n*-hexane (HX), ethyl acetate (EtOAc) and 2-methyltetrahydrofyran (2-MeTHF).

The green solvents employed in this work were chosen for their ability to extract both non polar (fatty acids) and polar (phenolic) compounds. The analysis of the concentration of the total phenolic compounds (TPC) and Radical Scavenging activity (RSA) highlighted significant differences among myrtle oils (Figure 1). ME and HX extracted oils showed a similar content of the TPC (0.45 ± 0.03 mg CA·g^−1^ oils and 0.33 ± 0.02 mg CA·g^−1^ oil, respectively).

By contrast, the TPCs of oils extracted with EtOAc (14.05 ± 0.87 mg CA·g^−1^ oil) and 2-MeTHF (8.87 ± 0.64 mg CA·g^−1^ oil) were considerably higher than that of ME oils. Similarly, on *Echium plantagineum* seeds, the extraction with EtOAc yielded an oil with a significantly higher TPC than the oil extracted with *n*-hexane [39]. The amount of phenolic compounds is affected by the polarity of the extraction solvents [8]. Our results further confirm this relationship; *n*-hexane is a solvent with a low polarity and has extracted a low amount of TPC while the bio-based solvents proposed in this work have a higher polarity and extracted a high concentration of the TPC. The Radical Scavenging Activity (RSA) results also showed a clear distinction between oils extracted with non-polar and polar solvents (Figure 1). ME and HX oils showed significantly lower RSA than those extracted with bio-based solvents.

#### 3.3.2. LC-ESI-Orbitrap-MS and LC-ESI-Orbitrap-MS/MS Analysis

The LC-ESI-Orbitrap MS profiles of the extracts obtained by myrtle oils revealed the presence of polyphenols mainly (Figure S1). The type and the relative concentration were different among the extracts; however, the analysis of the chromatograms (Figure S1), confirmed the TPC concentration results, that showed different amounts of TPC in the oils extracted with different methods.

29 major metabolites were detected and putatively identified on the basis of their accurate *m*/*z*, molecular formula, and mass fragmentation spectra in comparison with data present in the literature and databases, such as KNApSAcK, Mass Bank, and SciFinder (Table 2). The main compound groups present in myrtle seeds oils extracts included ellagitannins (such as galloyl hexose derivatives), flavonols (mainly myricetin, quercetin and kaempferol derivatives), phenolic acids, gallomyrtucommulones, and hydroxycinnamic derivatives. In addition, the lipophilic nature of the extract allowed the detection of some oxylipins, qualitatively present in each myrtle oil analyzed. The presence/absence of each metabolite in myrtle seed oil extract (HX, EtOAc, 2-MeTHF, ME) is reported in Table 2.

Compounds **1**, **2**, **3**, **7**, **8**, **12**, **17**, and **22** were identified as hydrolysable tannins. Most of them were previously described in *Myrtus communis* seeds by D’Urso, Sarais, Lai, Pizza and Montoro [32], with the exception of valoneic acid dilactone (**12**), attributed by comparison with literature mass spectrometric data. This metabolite was described in 2017 in *Myrtus nivelii* leaves by Rached, Bennaceur, Barros, Calhelha, Heleno, Alves, Carvalho, Marouf and Ferreira [41].

Most of the hydrolysable tannins were present in all oils, only ellagic acid (**17**) and its derivatives **7**, **8**, and **22** were not detected in ME and HX oils, probably due to the high polarity of these compounds. Ellagic acid, in our previous work, was suggested as the main responsible for the oxidative stability of myrtle hydro-alcoholic extracts over time, but it has been also hypothesized its pro-oxidant effect [16]. Ethylflavogallonate (**22**) was identified on the basis of mass fragmentation and molecular formula and comparison with literature data. This compound was previously reported in the stem bark of *Terminalia catappa* L. and was found to have interesting anti-inflammatory activity [44].

Compound **12** was previously reported in the leaves of *Myrtus nivelii* [41] and, in the present paper, was identified for the first time in seed oils extracted with EtOAc and 2-Me THF. Metabolites **4**, **6**, and **9** were identified, respectively, as galloylquinic acid, caffeoylhexose, and syringic acid. Galloylquinic acid and caffeoylhexose were previously described in myrtle seeds [32], whereas syringic acid was described in a previous paper in *Myrtus communis* leaves [40]. Galloylquinic acid was extracted with all the methods while the other compounds were present in some extracts only. Epigallocatechin (Compound **5**) was the only flavanol identified in the extracts under investigation, and it was recovered in EtOAc extracted oil only. Compounds **10**, **11**, **13**, **14**, **15**, **16**, **18**, **20**, **21**, **24**, and **25** were identified as flavonols. In particular, myrtle oils are characterized by derivatives of quercetin and myricetin. Quercetin derivatives are extracted indifferently with any method under investigation while myricetin derivatives were observed mostly in 2-MeTHF extract.

As observed in Figure S1, oils extracted with EtOAc and 2-MeTHF contain a higher concentration of hydrolysable tannins and flavonoids than those obtained with *n*-hexane and mechanically pressed oil. In these oils, tannins, and flavonoids have been also detected, but their concentration is so low to be considered negligible.

Compound **19** was identified as azelaic acid, a dicarboxyl acid described in 2017 by Ouchemoukh, Amessis-Ouchemoukh, Gómez-Romero, Aboud, Giuseppe, Fernández-Gutiérrez and Segura-Carretero [42] in Algerian honey, and here, identified for the first time in myrtle oil. This compound owns antimicrobial properties and has several pharmacological uses in dermatology, controlling facial acne, melasma, and rosacea [45]. Azelaic acid comes from the oxidative cleavage of oleic acid [46] and has been previously detected in other seed oils [47,48,49]. Compound **23** was identified as galloylmyrtucommulone C [32], present in almost all oils except those obtained with mechanical extraction. Myrtle oils extracted with EtOAc and 2-MeTHF share a similar phenolic profile but differ in the concentration of each phenolic compound, as revealed by the comparison of chromatograms (Figure S1). EtOAc myrtle oils are rich in galloylmyrtucommulone C (**23**), quercetin (**24**), and kaempferol (**25**) known in the literature for their high antioxidant activity [50,51], while 2-MeTHF oils contain high amounts of **24**, but less of **23** and **25**.

Compound **26** was attributed as terminolic acid, a triterpene acid present in all the oils obtained from myrtle seeds and previously described in *Myrtus communis* seeds [32].

Compounds **27**, **28**, and **29** were identified as oxylipins, hydroxyl fatty acids differing each other for the unsaturation degree and the number of hydroxyl groups. Oxylipins produced diagnostic MS/MS fragmentation patterns (reported in table) characterized by product ions generated by one or more consecutive neutral losses of 18 Da allowed us to ascertain the number of hydroxyl groups occurring in the oxylipin structure. As punctually described by D’Urso, Napolitano, Cannavacciuolo, Masullo and Piacente [43], oxylipins identified in Okra fruit, generate characteristic product ions, such as those at nominal *m/z* 171 (C_9_H_15_O_3_) and *m/z* 201 (C_10_H_17_O4), or those at nominal *m/z* 253 (C_15_H_25_O_3_), 229 (C_12_H_21_O_4_), 223 (C_14_H_23_O_2_), and 199 (C_11_H_19_O_3_), these hydroxyl groups could be located in the head (precisely at C9 and C10 positions) [43]. The present paper reports for the first time oxylipins in *Myrtus communis* seed oils. Oxylipins were detected in all the oils regardless of the extraction solvents or method used. These compounds, arising from the oxidation of PUFA, have been recently suggested as sensitive oxidation markers and have been proposed as food additives inhibiting fungal growth and mycotoxin production in food [52,53]. The extraction method affected the relative amount of oxylipins (Figure S1). In HX and ME oils, the 9-HODE (10,12) (**29**) was the most abundant, whereas in 2-MeTHF extracted oil the 9,10-DiHODE (**28**). By contrast, in EtOAc extracted oil, their amount was considerably lower as revealed by the analysis of the chromatogram. This difference might be due to a possible pro-oxidant effect of some antioxidant compounds. As already discussed above, it is known in the literature that some antioxidant compounds, depending on the concentration and under some particular conditions, may act as pro-oxidants, accelerating oil oxidation [51]. It is the case, for example, of α-tocopherol; in walnut oil subjected to accelerated storage conditions, the enrichment with α-tocopherol, over the concentration of 0.2%, decreased the oil oxidative stability and lead, at the same time, to the formation of oxylipins [54]. In the case of myrtle oil extracted with 2-MeTHF, it is possible to hypothesize for ellagic acid and its derivative a pro-oxidant effect similar to that observed for α-tocopherol in walnut oil.

#### 3.3.3. Quantitative Analysis of Myricetin and Its Derivatives

Preliminarily, ESI-QTrap-MS/MS spectra were analysed by the direct introduction of standard myricetin, myricetin-3-*O*-Galactopyranoside, and myricitrin at the dilution of 0.01 mg·mL^−1^ into the ESI source of a mass spectrometry instrument equipped with a triple quadruple analyser. The transitions of the ESI/MS/MS experiments analysis were recorded to develop a selective and sensitive UPLC-ESI-QTrap-MS/MS method using the technique through multiple reaction monitoring (MRM) mode.

The selected transitions were set on the tandem mass spectrometer as follows: myricetin: Precursor Ion 317 *m/z* to Product Ion 151 *m/z*; myricetin-3-*O*-galactoside: Precursor Ion 479 m/z to Product Ion 317; myricitrin: Precursor Ion 463 m/z to Product Ion 317.

Table 3 reports the quantitative results of the analysis of myricetin, myricitrin, and myricetin-3-*O*-Galactopyranoside in the polyphenolic extract obtained from oils from myrtle seeds performed with UHPLC-ESI-QTRAP-MS/MS analyses in MRM mode.

The quantitative analysis was possible only for Compounds **11**, **16**, and **21** because of their availability as commercial standards with necessary purity level for quantitative application. The results confirmed interesting amount of each compound, and they are in agreement with recently published quantitative results on myricetin derivatives in myrtle berries [55].

### 3.4. EPR Spin Trapping of Myrtle Seed Oils Subjected to Thermal Treatment

#### 3.4.1. Optimization of the Assay Temperature

Previous studies on the oxidative stability of plant extracts [16] and edible oils [37] highlighted the importance of the proper heating temperature for EPR oxidative stability experiments. This is the first time that an EPR oxidative stability test have been applied to myrtle seed oil, so oils were heated at 80, 90, and 100 °C to select the best temperature to perform the analysis. Figure S2 reports the evolution of the intensity of EPR signal of PBN adduct of myrtle seed oil extracted with the soxhlet method and heated inside the EPR cavity at 80, 90, and 100 °C. The results demonstrate that the rates of adduct formation increase with increasing temperatures. In oils heated at 100 °C, the production of radical adducts was fast from the beginning of the experiment while, when oils were heated at 80 or 90 °C, the production of PBN adducts was much slower. The effect of temperature on oil oxidation rates was studied on several oils heated at temperatures ranging from 110 to 140 °C, and it was described by the Arrhenius Equation [56]. In linseed oil, the effect of temperature on the formation of the PBN radical adduct was studied heating oil from 80 to 130 °C [57]. In that case, the peroxide values were used as oxidation index and were measured at different time intervals during oil heating. Increasing temperature significantly decreased the induction period (IP) and the maximum attainable peroxide value. Our results report a similar temperature effect on PBN adduct evolution over time; on myrtle seed oil heated at 100 °C, no IP could be determined while on oils heated at 90 or 80 °C, the IPs were, respectively, 4 and 68 min, calculated with the modified Boltzmann equation proposed in our previous paper [37]. In linseed oil, the IP values were halved at every 10 °C increase in temperature, whereas in myrtle oil, the IP reduction was much noticeable. Temperature also affects the maximum PBN-adduct signal intensity. In oil heated at 100 °C, the maximum PBN-adduct intensity was lower than that measured at 90 °C, whereas in myrtle oil heated at 80 °C, the maximum intensity was not reached even at the end of the experiment (ca. 200 min of continuous heating).

Based on these considerations, the temperature of 90 °C was employed to compare the oxidative stability of myrtle oils extracted with different methods.

#### 3.4.2. Thermal Treatment of Myrtle Oil

The spin trap PBN (*t*-butyl-α-phenyl nitrone) has been widely used to assess the oxidative stability of edible oils and alcohol containing matrices [16,37,58]. Figure S3 shows the EPR spectra of the radical species generated during the thermal treatment of soxhlet extracted oil at 80, 90, and 100 °C. No differences could be observed among spectra recorded at different temperatures; however, as already observed on sunflower oil, two PBN adducts are contemporaneously present [37]. Their simulation, carried out on spectra obtained after 61 min at 90 °C, provided the following results; the first spectrum, which accounts for the 65% of the radical species in heated myrtle oil, has hyperfine constants a_N_ 14.90 G; a_H_ 2.48 G; g 2.00573, whereas the other spectrum, which accounts for the 35% of the radicals present, is characterized by the following constants: a_N_ 14.70 G; a_H_ 2.88 G; g 2.00573. The EPR spectra considerably change until the first 11 min; then, no differences were observed among the oils extracted with different methods. Spectra recorded in the first 11 min highlighted some differences among myrtle oils. In oils extracted with cold *n*-hexane and 2-MeTHF, the first spectra were quite different compared to the next spectra as shown in Figure S4. In these oils, the radical species with a_N_ 14.95 G; a_H_ 4.65 G; g 2.0057 (*n*-hexane extracted oils) and a_N_ 14.95 G; a_H_ 4.30 G; g 2.00555 (2-MeTHF) were observed in the first minute of heating and disappeared thereafter. This species has been already detected in sunflower oil and has been identified as the adduct of MNP (2-methyl-2-nitrosopropane), a decomposition product of PBN-OOR adduct [37]. In ME and EtOAc extracted oils, no MNP adduct was detected since the first spectra were simulated with the following hyperfine constants: a_N_ 14.85 G; a_H_ 2.20 G; g 2.00593 (ME oil) and a_N_ 14.89 G; a_H_ 2.30 G; g 2.00599 (EtOAc extracted oil).

#### 3.4.3. Thermal Treatment of Myrtle Oils Obtained with Mechanical and Chemical Methods

**Figure 2:**
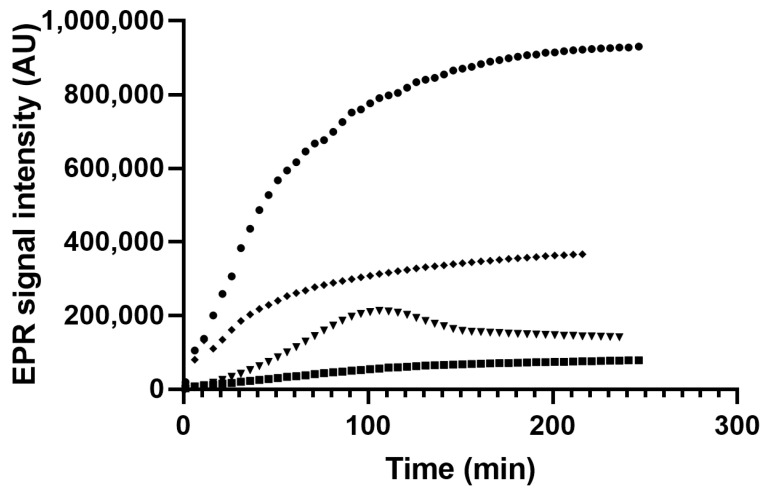
Evolution of EPR intensity of the PBN adduct of myrtle seed oil extracted with *n*-hexane (●), ethyl acetate (■), 2-methyl tetrahydrofuran (♦), and Mechanical extraction (▼) and heated (PBN 125 mM final concentration) at 90 °C. Each point is the mean of two measures.

Figure 2 reports the evolution of the intensity of PBN-adduct as a function of time, of myrtle oils extracted with mechanical and chemical methods, and heated at 90 °C. The kinetic patterns of PBN adducts demonstrate that the extraction method affects myrtle oils oxidation rates. In oils extracted with cold *n*-hexane, the PBN adduct intensity was high from the beginning of the experiment and kept on growing with time reaching the highest intensity (about 900,000 AU) at the end of the experiment after about 200 min of continuous heating. By contrast, EtOAc and 2-MeTHF extracted oils showed a rate of PBN adduct formation considerably lower. In particular, in oils extracted with EtOAc the formation of PBN adducts was low even after 200 min of continuous heating at 90 °C. The thermal treatment of 2-MeTHF oil determined a PBN adduct intensity intermediate between HX and EtOAc oils, whereas in ME oils, the PBN adduct increased up to 110 min then slightly decreased and reached an equilibrium after 150 min of warming up. The kinetic curve of the ME oil recalls those previously observed on sunflower and olive oils which can be fitted by a double sigmoidal curve [37]. Both on sunflower and olive oils the decay of the signal intensity was explained as result of the equilibrium between the formation and the decomposition of the PBN adduct. Cold pressed and refined oils, like extra virgin and sunflower oils, own low amounts of free fatty acids, whereas their amounts are considerably higher in unrefined oils. Some studies highlighted the role of free fatty acids in bulk oils oxidation pointing out their pro-oxidant effect, the ability to catalyze hydroperoxide cleavage, and the ability to react with transition metals [59]. In stripped extra virgin oil stored for 20 days at 60 °C and added with increasing amounts of purified FFA, Paradiso, Pasqualone, Summo and Caponio [59] highlighted a dose dependent and a time related effect of FFA on oil oxidation. Low doses of FFA were responsible, in the first stages of oxidation, for an increase in hydroperoxide formation followed by a hydroperoxide decrease due to their decomposition. By contrast, high FFA concentrations favored, as oxidation went on, alternative pathways involving the oxidation of FFAs, even those less liable to oxidation that overlapped with the hydroperoxide decomposition and with the oxidation of esterified fatty acids.

Regarding myrtle, oils with low FFA concentration (ME oil) showed an increase in PBN adduct intensity followed by a decrease that was not observed in myrtle oils with high FFA concentration (see Table 1). In the oils tested in this study, it is not possible to define the role of FFA on the intensity of PBN-adduct as a function of time since, when oils are heated at the same temperature, the kinetic curves are affected by different factors. Besides FFA concentration, the unsaturation degree of the oils and the antioxidants concentration affect the generation rates of PBN-adducts and thus the kinetic curve shapes. Fatty acids own different sensitiveness to thermal oxidation depending on the number and the position of the double bonds present within the molecule [57]. Moreover, unbound fatty acids are more liable to oxidation than fatty acids in triacylglycerol molecules. The antioxidants present in the oils inhibit fatty acids oxidation by scavenging peroxyl radicals and breaking off the propagation of the radical chain reactions [56].

The oils extracted from myrtle seeds with different methodologies share a similar fatty acid concentration but differ for the amounts of FFA and antioxidant compounds. Myrtle oil extracted with *n*-hexane (cold extraction) owns very few phenolic compounds (see Figure 1) and a concentration of FFA intermediate compared to the other myrtle oils (see Table 1). In this case the phenolic compounds present might not be sufficient to counteract the generation of PBN adducts (or to compete with the spin trap in the reaction with radicals), leading to a prompt and sudden rise of the PBN adduct intensity. By contrast, EtOAc-extracted myrtle oil has a phenolic compounds concentration more than three-fold higher than HX oils and a higher FFA concentration. Despite the high FFA concentration, the intensity of the PBN adduct was low during the experiment time. The phenolic compounds present in the oil might have broken off the propagation of the radical chain reaction decreasing the amount of radicals available for the entrapment by PBN.

In 2-MeTHF extracted oils, the intensities of PBN adduct over time was intermediate between those in HX and EtOAc extracted oils. The extraction with 2-MeTHF provided an oil with a concentration of free and esterified fatty acids similar to the EtOAc extraction and a concentration of phenolic compounds significantly lower. Therefore, considering the concentration of FFA, the PBN adduct intensity is lower than that it would have expected maybe due to the presence of high amounts of antioxidants capable of reacting with lipid radicals and hindering the formation of the PBN adduct. The relationships among PV, TPC, and FFA will be further developed in the next paragraph. Besides the TPC concentration, the type of the antioxidants extracted may have a role in oils oxidative stability. The myrtle oils with the highest thermal oxidative stability (EtOAc and 2-MeTHF extracted oil) share a similar phenolic profile but differ in the concentration of each phenolic compound, as revealed by the comparison of chromatograms (Figure S1). The possible role of some phenolic compounds in myrtle oils oxidative stability, measured as AUC, will be further discussed in the paragraph of multivariate analysis.

#### 3.4.4. Relationship between EPR Intensity and Composition of Oils Subjected to Thermal Treatment

In our previous work on extra virgin olive and sunflower oils, we fitted the experimental curve EPR intensity vs. time with a modified Boltzmann equation considering the slope of the first part of the curve before the sudden increase of the PBN adduct intensity [37]. In that case, the oxidative stability of the oils was expressed as lag time. On myrtle oils it was possible to calculate the lag time only in ME oils. For the other oils the only significant parameter, which could be extracted from the curves representing the EPR intensity of the PBN adduct vs. time, is the area under the curve (AUC). The AUC value for each myrtle oil is reported in Table 1. As previously discussed, the extraction method affects the kinetic patterns of PBN adducts vs. time, and hence the AUC. The kinetic pattern of PBN adduct is the result of the equilibrium of different factors; in fact, it can be hypothesized that the AUC is influenced by (i) the peroxide value (PV), since the decomposition of peroxides generates free radicals; (ii) the antioxidant amount, measured as total phenolic compounds (TPC), since antioxidants decrease the amount of free radicals trapped by PBN; and (iii) the amount of FFA, since these compounds are those more susceptible of oxidation [59].

Based on this hypothesis, an experimental relationship linking AUC, PV, TPC, and FFA has been found for myrtle seeds oils:

AUC (a.u.)/1 × 10^6^ = 1.62 × PV − 12.78 × TPC − 2.44 × FFA (1)

This relationship worked very well for solvent extracted oils but not for the mechanically obtained one, as observed in Figure S5 that reports the experimental and calculated values of AUC. This fact may not be surprising considering that ME oil shows a different shape of the kinetic curve (see Figure 2), so other factors besides those previously described may affect the kinetic pattern of PBN adduct and AUC. In myrtle seed oils extracted with solvents, the shape is completely different and the EPR intensity continuously increases from the beginning of the experiment without reaching a maximum after 200 min. Further studies are needed to explain the shape of the kinetic curve and the relationship between the parameters considered in the model (PV, TPC, and FFA) for solvent extracted myrtle seed oils.

### 3.5. Multivariate Data Analysis

Multivariate data analyses were adopted to classify myrtle oils on the basis of their phenolic profile to reveal the differences among groups of metabolites extracted in an untargeted manner. Multivariate data analysis was basically performed following the experimental protocol described by D’Urso et al. and Crescenzi et al. [35,60]. LC-ESI-Orbitrap-MS chromatograms were pre-processed using MZmine (a free software) to recompense variations in retention time and *m/z* value between different chromatographic runs. A peak list table was obtained from pre-processed chromatograms. In the final matrix produced, rows represented the individual samples (36 samples: 12 biological samples in technical duplicates) and columns represented integrated and normalized peak areas. These data were subjected to untargeted analysis, analyzing them with an unsupervised multivariate data analysis method (PCA).

**Figure 3:**
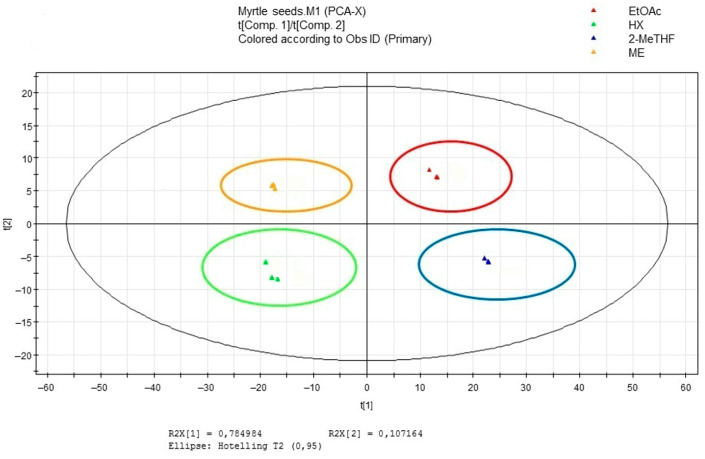
Multivariate Data Analysis results for samples extracted with different methods. LC-MS data were fused with RSA, PV, TPC and EPR data results. PCA Score scatter Plot.

Principal component analysis (PCA) was achieved by applying the peak area of each peak observed in the LC-MS dataset (excluding the noisy). In addition, four variables were added to the matrix obtained by MZmine elaboration, corresponding, respectively, to Peroxide value (PV), Total Phenolic (TPC), radical scavenging activity (DPPH), and EPR spin Trapping AUC values, measured for each sample in previous experiments described in the present paper. The integration of the data obtained by different analytical strategies in metabolomics can give very informative results when analyzed with a multivariate data analysis approach [61]. The resulting score scatter plot is reported in Figure 3. The first component describes the 78.5% of variance while the second the 10.72%. The choice of principal components was realized on the basis of the fitting (R2X) and predictive (Q2X) values for the PCA model. The score scatter plot in Figure 3 A is colored according to the extraction methods applied to myrtle seeds. It is clear there is a discrimination of the samples based on this parameter; in fact, the cluster of each extraction method is confined in a different quadrant of the score scatter plot. Thus, this is evidence that metabolites extracted present evaluable differences. With the analysis of the loading scatter plot (Figure S6A), it was possible to investigate the variables responsive for the observed clustering. Phenolic compounds like hydrolyzed tannins, hydroxycinnamic acids, and some flavonols are concentrated in the high right part of the plot, characterizing mainly samples extracted with ethyl acetate. By coloring the plot with the aim to make evident the variables not taken with LC-MS analysis (Figure S6B), it is possible to observe that the higher value for TPC, PV, and RSA results are concentered in the high right plot, the one characterized by the most intense presence of polyphenolic metabolites. The AUC that has been used in this paper to estimate the oxidative stability of myrtle oils fall in the left down quadrant, characterized in the score scatter plot by samples extracted with *n*-hexane and in the loading scatter plot by a reduction in the presence of metabolites, with the exception of some derivatives of myricetin. This separation makes us suppose that myricetin aglicone and its derivatives, due to their high antioxidant properties, could be the main party responsible for myrtle oils’ oxidative stability.

A recent paper demonstrated that the addition of exogenous myricetin effectively improved the thermal oxidation stability of *Eucommia ulmoides* L. seed oil, extended its oxidation induction time, and increased the stability of the oil [62]. According to these authors, the protective effect of myricetin is the result of its stability in the lipid dispersion, the antioxidant activity, and its oxygen barrier ability [62]. Based on these results, EtOAc is the most interesting solvent for the extraction of polyphenols from myrtle seeds, by-products of the myrtle liqueur production.

## 4. Conclusions

In this paper, for the first time, myrtle seeds obtained from the by-products of the myrtle liqueur processing industry have been used to extract an oil that might be employed in food or the cosmetic industry. A sustainable and green approach was followed in the extraction process. A mechanical and solvent extraction methods using bio-based, non-toxic, and biodegradable solvents were compared to the *n*-hexane extraction, which is the benchmark for oil extraction. The biobased solvents (EtOAc and 2-MeTHF) employed in this work are characterized by different polarity that affected the concentration of phenolic compounds but had little effect on oils fatty acids composition. The results demonstrate that the extraction process significantly affects the oil quality in terms of PV, profile and total amount of phenolic compounds, FFA, and oxidative stability, but it has little effect on fatty acids composition. All myrtle oils analyzed, in fact, have a similar fatty acid composition with a concentration of linoleic acid of about 77% of the fatty acids. The multivariate approach applied to classify myrtle seed oils on the basis of their phenolic profile provides a clear discrimination of the samples extracted with different methods. Depending on the phenolic profile and thus on the extraction method, myrtle oils could find different applications. The integration of data arising from different analytical tools in a multivariate platform was very helpful to identify the relationships among data. In this paper the oxidative stability of myrtle oil was studied with the spin trapping method coupled with EPR spectroscopy. This work has further highlighted the potential of this technique in the study of the oxidative stability of oils. The kinetic curves representing the EPR intensity of the PBN adduct vs. time are significantly affected by the chemical composition of the oils; in particular, FFA, TPC, and the phenolic profile seem to have a role in the evolution of radical species over time. The combination of EPR and LC-MS data in a multivariate data analysis highlighted the importance of myricetin and its derivatives in the oxidative stability of myrtle oils. Myricetin and its derivatives are among the strongest antioxidants found in myrtle seed oils extracted in this work since contain in their structure a pyrogallol moiety. The role of these flavanol compounds in oils oxidative stability certainly needs further study. Moreover, a mathematical relationship linking the oils oxidative stability measured as AUC and some parameters like PV, TPC, and FFA was found. This model fitted very well for solvent extracted oils but not for the ME oil, so further studies are needed to identify other parameters that may be involved in the kinetic pattern of PBN adduct and AUC.

In conclusion, some consideration about myrtle seed oil and the methods used to extract it can be drawn. The mechanical extraction, proposed for the first time in this paper for myrtle seed oil, provided an oil with a low concentration of FFA and a low PV as compared to the other extraction methods. This oil, despite having a low content of phenolic compounds and a low antioxidant capacity, has a higher oxidative stability than oils extracted with *n*-hexane, as demonstrated by the EPR analysis, which makes it a good candidate for food or cosmetic applications. Even the oil extracted with EtOAc could be proposed for future applications. It is an oil with a high content of phenolic compounds, in particular myricetin and its derivatives, that might contribute to its high oxidative stability and its high antioxidant activity. This oil, in fact, despite the high concentration of PV and FFA, has the highest oxidative stability as pointed out by the low AUC.

## Figures and Tables

**Table 1:** Oil Yield, peroxide values (PV), Area Under the of the kinetic Curve (AUC) of the EPR intensity of the PBN adduct vs. time, and Fatty Acid percentage composition of the myrtle oils obtained with chemical and mechanical extraction methods.

	Oil Extraction Methods			
	ME	HX	2-MeTHF	EtOAc
Chemical and spectroscopic parameters				
Yield ^y^	3.21 ± 0.27 b	9.37 ± 0.62 a	10.55 ± 2.82 a	5.41 ± 0.42 b
PV ^z^	99.91 ± 4.47 b	99.51 ± 5.53 b	121.38 ± 6.45 a	126.96 ± 3.89 a
AUC	2.9 × 10^7^ ± 5.5 × 10^6^ c	1.5 × 10^8^ ± 1.3 × 10^7^ a	6.8 × 10^7^ ± 1.3 × 10^7^ b	9.4 × 10^6^ ± 4.4 × 10^6^ c
Fatty acids (%)				
C16:0	7.5 ± 0.1 a	7.5 ± 0.2 a	7.5 ± 0.3 a	7.4 ± 0.3 a
C18:0	3.3 ± 0.1 a	3.3 ± 0.1 a	3.3 ± 0.1 a	3.2 ± 0.2 a
C18:1	9.2 ± 0.2 a	8.8 ± 0.3 a	8.9 ± 0.5 a	9.0 ± 0.1 a
C18:2	78.0 ± 0.1 a	77.3 ± 0.4 ab	76.7 ± 0.6 b	77.8 ± 0.2 a
FFA	1.2 ± 0.1 c	3.1 ± 0.9 b	6.5 ± 0.5 a	7.0 ± 0.6 a

^y^ yield was expressed as percentage; ^z^ Peroxide values were expressed as µ equivalents oxygen g^−1^. Within each row, different lower case letters relate to significant differences among extraction methods. Differences were calculated according to Tukey’s test (*p* ≤ 0.05).

**Table 2:** LC-ESI-Orbitrap-MS profiles of Myrtle seeds oils polyphenolic extract. The oils from myrtle seeds were obtained with different extraction methods: EtOAc: ethyl acetate; HX: cold *n*-hexane; 2-MeTHF: 2-methyl tetrahydrofuran; ME: mechanical extraction.

Peak N°	RT	[M-H]^−^	MS/MS	Molecular Formula	Δppm	Identity	EtOAc	HX	2-MeTHF	ME	Ref.
1	1.95	331.0667	313/271/169	C_13_H_16_O_10_	2.22	monogalloyl-hexose	x	x	x	x	[32]
2	2.04	481.0617	301/275	C_20_H_18_O_14_	1.01	HHDP-glucose	x	x	x	x	[32]
3	3.01	633.0726	301/421/615	C_34_H_22_O_22_	1.1	strictinin (DigalloylHHDP glucose)	x	x	x	x	[32]
4	3.71	343.0667	191/169	C_14_H_16_O_10_	1.9	galloylquinic acid	x	x	x	x	[32]
5	4.37	305.0660	174/270	C_15_H_14_O_7_	1.9	epigallocatechin	x	-	-	-	[32]
6	7.54	341.0881	161/179	C_15_H_17_O_9_	4.16	caffeoylhexose	-	-	x	-	[32]
7	8.4	785.0836	301/433/766	C_34_H_26_O_22_	0.21	tellimagrandin I	x	-	x	-	[32]
8	12.3	463.0511	301	C_20_H_16_O_13_	1.2	ellagic acid hexoside	x	-	x	-	[32]
9	13.96	197.0455	167.89	C_9_H_10_O_5_	5.7	syringic acid	x	-	x	x	[40]
10	14.6	631.0948	479/317	C_28_H_24_O_17_	2.9	myricetin galloyl hexoside	x	-	x	-	[32]
11	15.35	479.0835	317	C_21_H_20_O_13_	3.2	myricetin-3-*O*-gal	-	-	x	-	[32]
12	16.65	469.0056	425	C_21_H_10_O_13_	4.01	valoneic acid dilactone	x		x		[41]
13	17.99	449.0730	317	C_20_H_18_O_12_	3.6	myricetin pentoside	x	-	x	-	[32]
14	18.2	615.0985	463/301	C_28_H_24_O_16_	0.53	quercetingalloyl-hexoside	x	x	x	x	[32]
15	18.6	463.0875	301	C_21_H_20_O_12_	0.9	quercetinhexoside	x	x	x	x	[32]
16	18.7	463.0875	317	C_21_H_20_O_12_	0.9	myricitrin	x	x	x	x	[32]
17	20.35	300.9990	257.00/228.99/185.07	C_14_H_6_O_8_	3.7	ellagic acid	x		x	-	[32]
18	21.6	447.0926	301	C_21_H_20_O_11_	1.4	quercetindeoxy-hexoside	x	x	x	x	[32]
19	21.68	187.0977	125.03	C_9_ H_16_ O_4_	6.6	azelaic acid	x	x	x	x	[42]
20	23.2	615.0984	317/463	C_28_H_24_O_16_	0.7	myricetingalloyl-deoxyhexose	x	x	x	x	[32]
21	23.53	317.0302	178.95/151.04	C_15_ H_10_ O_8_	3.2	myricetin	x	-	x	-	[32]
22	23.99	497.0363	301.05	C_23_ H_14_ O_13_	2.5	ethylflavogallonate	x	-	x	-	[32]
23	24.68	567.2082	271.03/313.11/211.12/169.03	C_27_ H_36_ O_13_	1.8	gallomyrtucommulone C	x	x	x	-	[32]
24	27.05	301.0354	178.86/150.88	C_15_ H_9_ O_7_	3.9	quercetin	x	x	x	x	[32]
25	30.24	285.0403	150.95	C_15_H_10_O_6_	3.4	kaempferol	x	x	x	x	[32]
26	31.02	503.3374		C_30_H_48_O_6_	1.36	terminolic acid (triterpene acid)	x	x	x	x	[32]
27	36.6	313.2382	295/277/195/ 183/129	C_18_H_34_O_4_	2.8	9,10-DiHOME	x	x	x	x	[43]
28	37.9	311.2225	293/275/211/ 201/183/171	C_18_H_32_O_4_	2.7	9,10-DiHODE	x	x	x	x	[43]
29	40.82	295.2275	277/195/179/ 171	C_18_H_31_O_3_	2.6	9-HODE (10,12)	x	x	x	x	[43]

**Table 3:** Quantitative results of myricetin, myricitrin and myricetin-3-*O*-galactopyranoside in the polyphenolic extract obtained from oils from Myrtle seeds. Analyses performed by UHPLC-ESI-QTRAP-MS/MS analyses in MRM mode.

Ref.	Compounds	EtOAc (mg·g^−1^ ± SD)	HX (mg·g^−1^ ± SD)	2-MeTHF (mg·g^−1^ ± SD)	ME (mg·g^−1^ ± SD)
11	Myricetin-3-*O*-gal	0.12 ± 0.02	0.01 ± 0.00	1.82 ± 0.02	0.11 ± 0.05
16	Myricitrin	4.16 ± 0.17	4.67 ± 0.15	4.59 ± 0.05	4.67 ± 0.19
21	Myricetin	1.65± 0.00	0.06 ± 0.02	1.46± 0.06	0.13 ± 0.09

## Data Availability

The data are contained within the article and supplementary materials.

## Supplementary Materials

The following supporting information can be downloaded at: https://www.mdpi.com/article/10.3390/antiox12010154/s1, Figure S1: LC-ESI-Orbitrap-MS profiles of phenolic compounds extracted from myrtle seed oils; Figure S2: Evolution of EPR intensity of myrtle seed oil extracted with hexane (soxhlet) and heated with PBN; Figure S3: EPR spectra of myrtle oils extracted with solvent and mechanically pressed extraction methods; Figure S4: Experimental and simulated EPR spectra of myrtle oils extracted with solvent and cold pressed extraction methods; Figure S5: Experimental and calculated AUC values determined for myrtle oils extracted with solvents; Figure S6: Multivariate Data Analysis results: PCA Loading Scatter Plot.
