# Supplementary material for: Sustainable Extraction Methods Affect Metabolomics and Oxidative Stability of Myrtle Seed Oils Obtained from Myrtle Liqueur By-Products: An Electron Paramagnetic Resonance and Mass Spectrometry Approach
Source: Antioxidants (Basel). 2023 Jan 9;12(1):154. doi: 10.3390/antiox12010154 (PMC9854790; doi:10.3390/antiox12010154)
Supplement: Supplementary file 1 [file antioxidants-12-00154-s001.zip › antioxidants-2064312-supplementary.pdf]

Sustainable Extraction Methods Affect Metabolomics and Oxidative Stability of Myrtle Seed Oils Obtained from Myrtle Liqueur By-Products: An Electron Paramagnetic Resonance and Mass Spectrometry Approach

Angela Fadda [1,*,†], Paola Montoro [2,†], Gilda D'Urso [2], Nicoletta Ravasio [3], Federica Zaccheria [3] and Daniele Sanna [4]

[1] Institute of the Sciences of Food Productions, National Research Council, Traversa La Crucca, 3, 07100 Sassari, Italy
[2] Department of Pharmacy, University of Salerno, Via Giovanni Paolo II 132, 84084 Fisciano, Italy
[3] Institute of Chemical Sciences and Technologies "G. Natta", National Research Council, Via Golgi 19, 20133 Milano, Italy
[4] Institute of Biomolecular Chemistry, National Research Council, Traversa La Crucca, 3, 07100 Sassari, Italy
* Correspondence: angela.fadda@cnr.it; Tel.: +39-079-284-1714
† These author contributed equally to this work.

SUPLEMENTARY INFORMATION FOR PUBLICATION

- Figure S1: LC-ESI-Orbitrap-MS profiles of phenolic compounds extracted from myrtle seed oils obtained with different techniques: (A) ME; (B) EtOAc; (C) 2-MeTHF; (D) HX.
- Figure S2: Evolution of EPR intensity of myrtle seed oil extracted with hexane (soxlet) and heated with PBN (125 mM final concentration) at 80 (■), 90 (●) and 100 (▲) °C. Each point is the mean of two measures.
- Figure S3: EPR spectra of myrtle oils extracted with solvent and mechanically pressed extraction methods. (A) Spectra of myrtle oil extracted with soxhlet method and heated for 61 minutes at 353, 363 and 373 K; (B) experimental (solid line) and simulated (dotted line) spectra of of myrtle oil extracted with soxhlet method and heated for 61 minutes at 363 K; (C) spectra of myrtle oil extracted with n-hexane and heated at 363 K for 1, 6, 11 minutes; (D) experimental (solid line) and simulated spectra (dotted line) of myrtle oils extracted with *n*-hexane and heated at 363 K for 1 minute.
-Figure S4: EPR spectra of myrtle oils extracted with solvent and cold pressed extraction methods. (A) Spectra of myrtle oil extracted with 2-MeTHF and heated at 363 K for 1, 6, 11, 16 and 21 minutes; (B) experimental (solid line) and simulated (dotted line) spectra of myrtle oil extracted with 2-MeTHF and heated for 1 minute at 363 K; (C) experimental (solid line) and simulated (dotted line) spectra of myrtle oil obtained with mechanical extraction and heated for 1 minute at 363 K; (D) experimental (solid line) and simulated spectra (dotted line) of myrtle oil extracted with ethyl acetate and heated at 363 K for 1 minute.

- Figure S5: Experimental and calculated AUC values determined for myrtle oils extracted with with HX (●), 2-MeTHF (◆), EtAoC (■)
- Figure S6: Results of Multivariate Data Analysis: (A) PCA Loading Scatter Plot; (B) PCA Loading Scatter Plot with in colour evidence of the fused data.

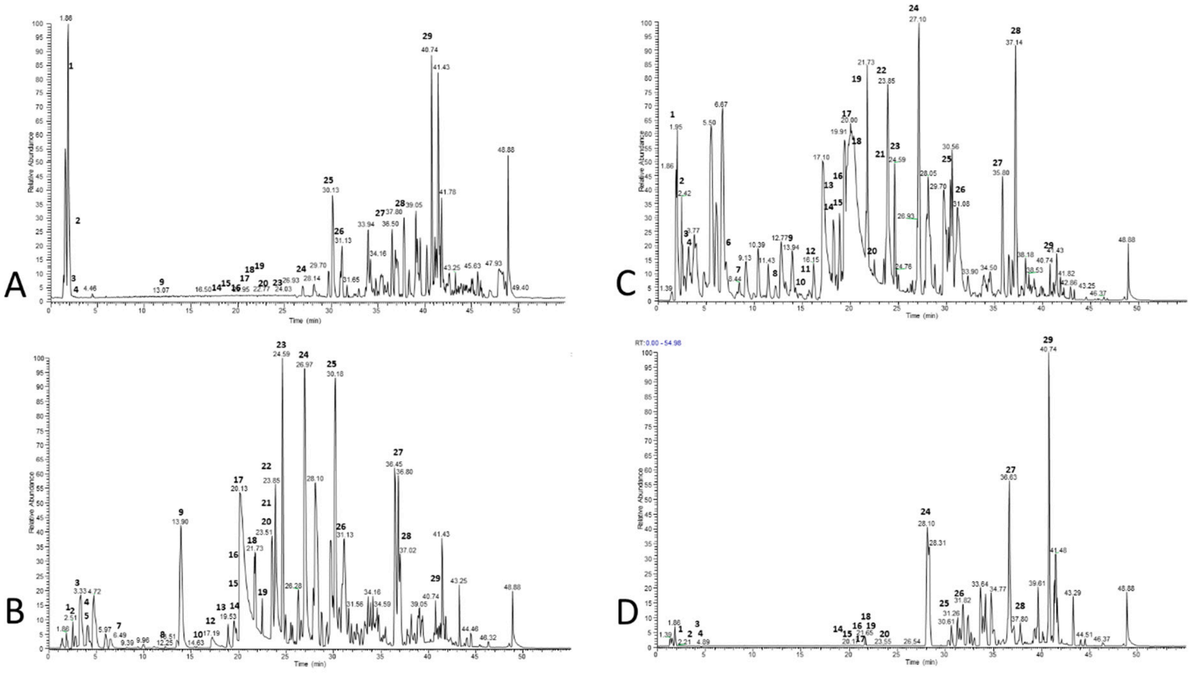

Figure S1. LC-ESI-Orbitrap-MS profiles of phenolic compounds extracted from myrtle seed oils obtained with different techniques: (A) ME; (B) EtOAc; (C) 2-MeTHF; (D) HX.

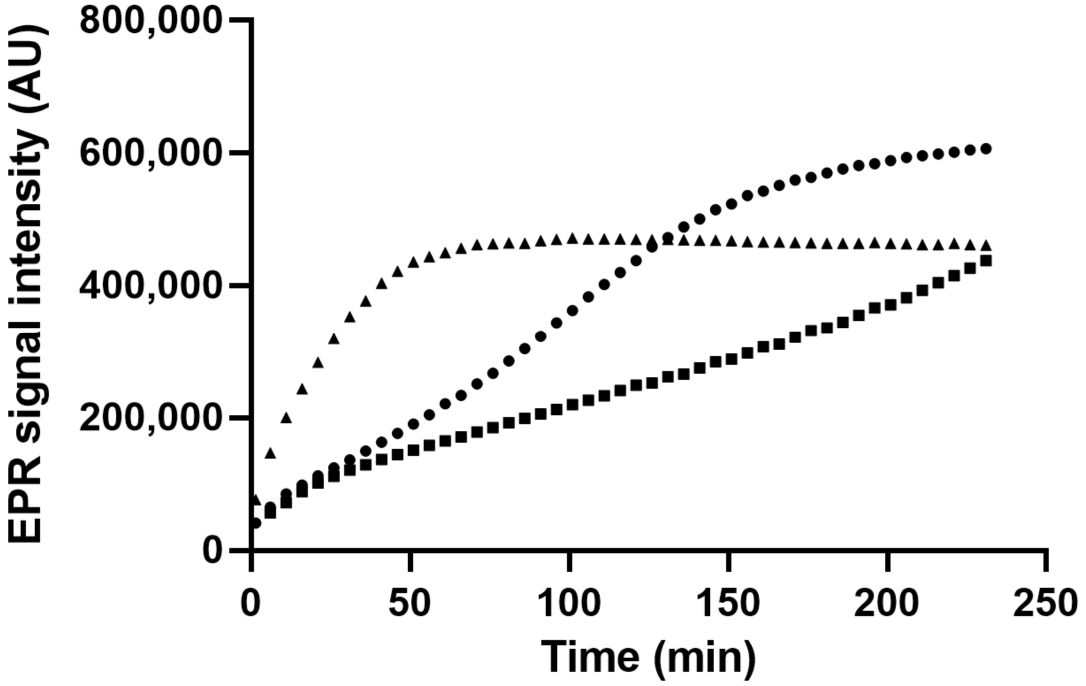

Figure S2. Evolution of EPR intensity of myrtle seed oil extracted with hexane (soxlet) and heated with PBN (125 mM final concentration) at 80 (■), 90 (●) and 100 (▲) °C. Each point is the mean of two measures

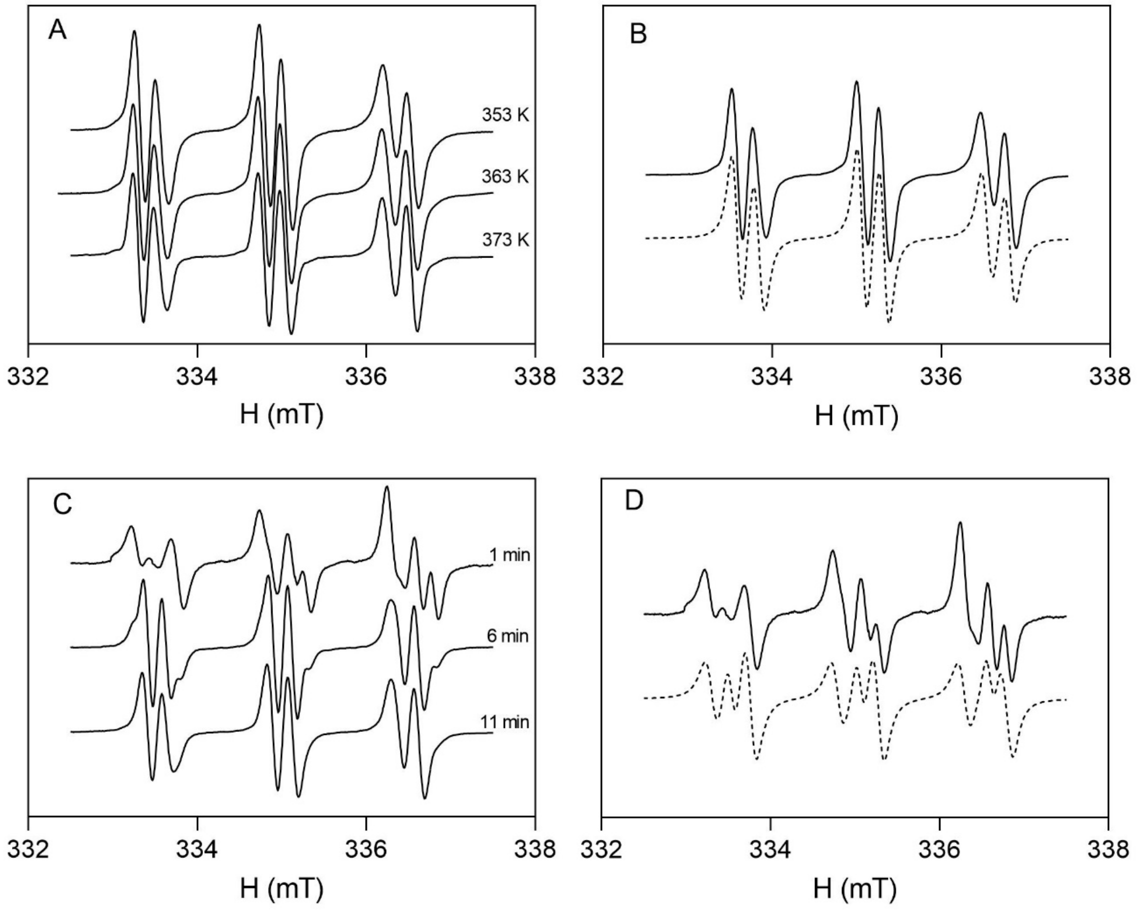

Figure S3. EPR spectra of myrtle oils extracted with solvent and mechanically pressed extraction methods. (A) Spectra of myrtle oil extracted with sohxlet method and heated for 61 minutes at 80, 90 and 100 °C; (B) experimental (solid line) and simulated (dotted line) spectra of of myrtle oil extracted with sohxlet method and heated for 61 minutes at 90°C; (C) spectra of myrtle oil extracted with n-hexane and heated at 90 °C for 1, 6, 11 minutes; (D) experimental (solid line) and simulated spectra (dotted line) of myrtle oils extracted with n-hexane and heated at 90 °C for 1 minute.

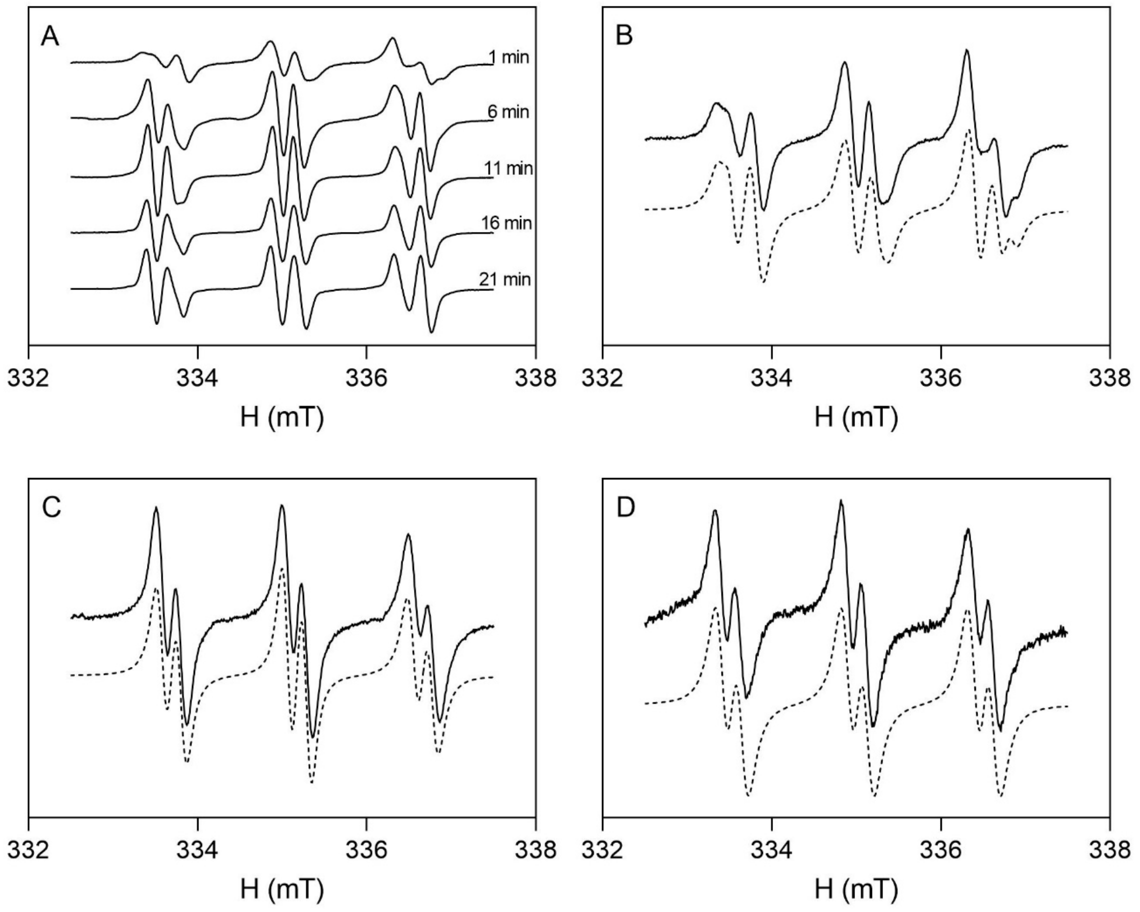

Figure S4. EPR spectra of myrtle oils extracted with solvent and cold pressed extraction methods. A) Spectra of myrtle oil extracted with 2-MeTHF and heated at 90 °C for 1, 6, 11, 16 and 21 minutes; B) experimental (solid line) and simulated (dotted line) spectra of myrtle oil extracted with 2-MeTHF and heated for 1 minute at 90 °C; C) experimental (solid line) and simulated (dotted line) spectra of myrtle oil obtained with mechanical extraction and heated for 1 minute at 90 °C; D) experimental (solid line) and simulated spectra (dotted line) of myrtle oil extracted with ethyl acetate and heated at 90 °C for 1 minute.

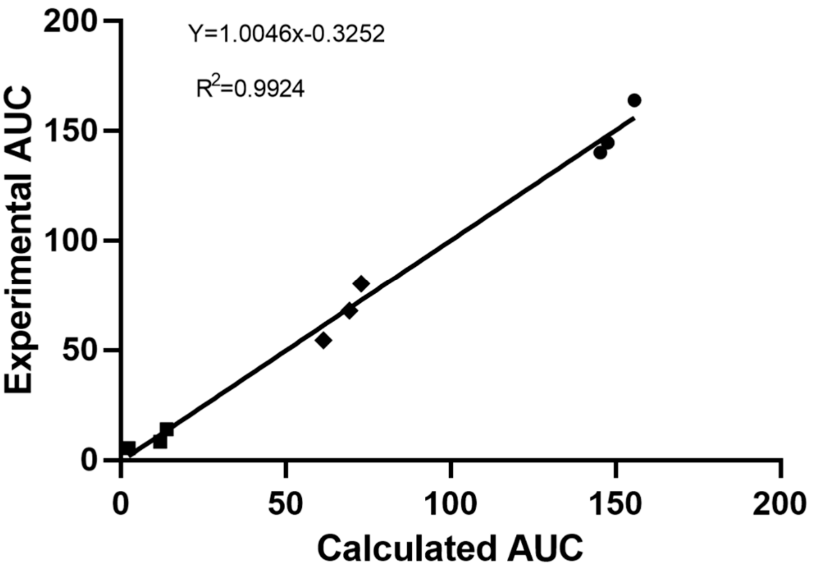

Figure S5. Experimental and calculated AUC values determined for myrtle oils extracted with HX (●), 2-MeTHF (◆), EtAoC (■).

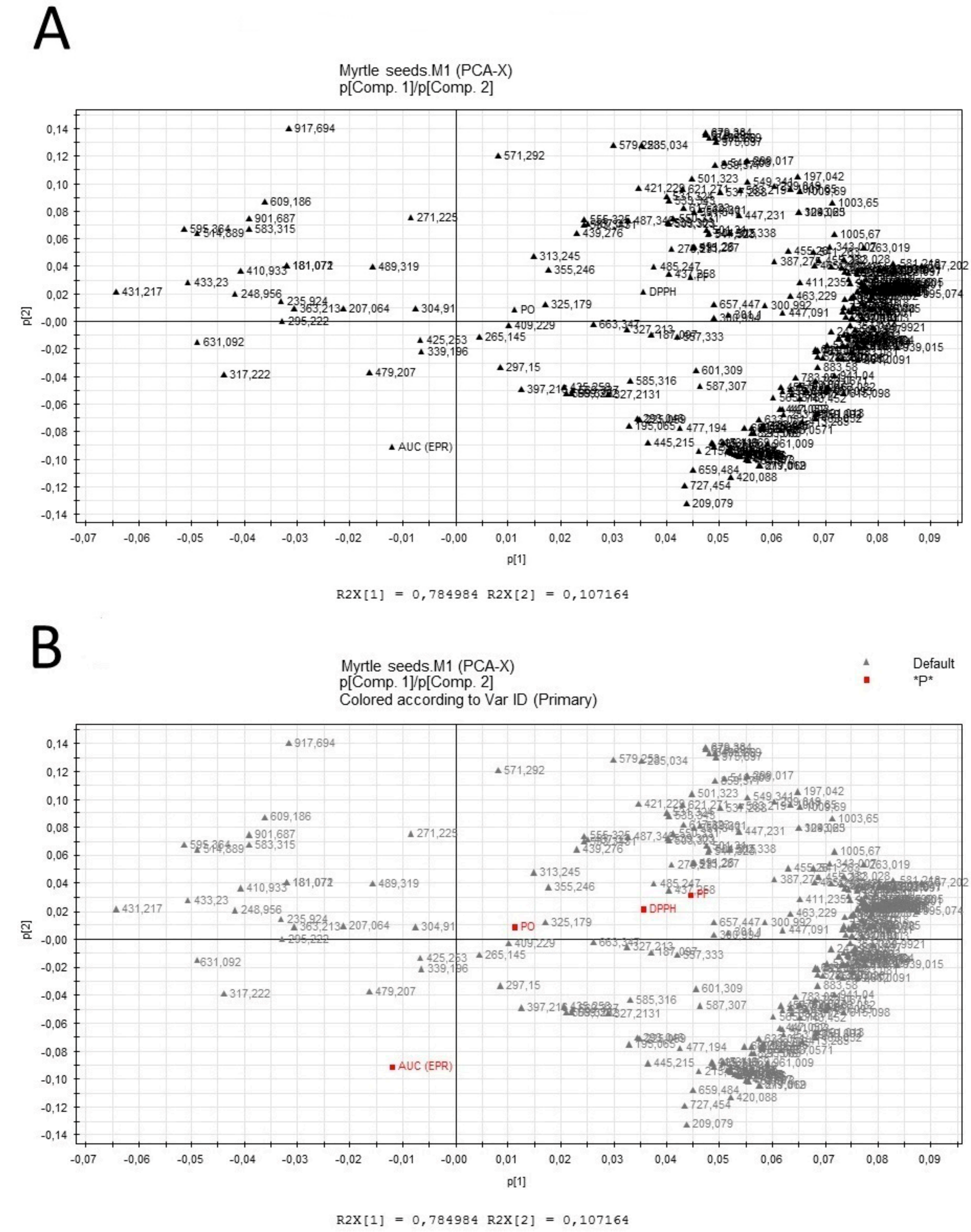

Figure S6. Results of Multivariate Data Analysis for samples extracted with different methods. LC-MS data were fused with DPPH, PO, PF and EPR data results. (A) PCA Loading Scatter Plot; (B) PCA Loading Scatter Plot with in colour evidence of the fused data
